# Isolated unilateral duplication of vas deferens, a surgical enigma: a case report and review of the literature

**DOI:** 10.1186/1757-1626-2-167

**Published:** 2009-10-26

**Authors:** Chintamani, Rohan Khandelwal, Megha Tandon, Yashwant Kumar

**Affiliations:** 1 Department of Surgery, Vardhman Mahvir Medical College & Safdarjang Hospital, New Delhi, 110023, India

## Abstract

**Background:**

Duplication of vas deferens is a very rare congenital anomaly with only a few cases reported in the literature. Injury to vas deferens is a recognized complication during hernia surgery and the possibilities of injury may increase with lack of awareness of this anomaly.

**Case report:**

A-31-year old gentleman with unilateral duplication of vas deferens that was diagnosed during right sided open hernia surgery is reported. The patient had undergone surgery for left inguinal hernia one year previously at the same center and no such anomaly was detected. During intra-operative examination of scrotum, the testes and epididymis were found to be normal. Patient had an uneventful recovery from hernia surgery.

**Conclusion:**

Unilateral duplication of vas deferens is rare. The identification of this anomaly is essential in order to minimize the incidence of injury to vas during hernia surgery. This case reinforces the importance of routine identification of vas deferens and awareness about this anomaly during inguinal hernia surgery.

## Background

Duplication of vas deferens(VD) is rare with only a few cases reported in the world literature [1-6]. A large number of hernia surgeries are performed world over, annually and injury to VD is a recognized complication of this surgery. Identification of VD during dissection of the hernia sac is therefore considered mandatory. The chances of iatrogenic injury to the vas are increased if this anomaly is not detected making it essential for a surgeon to be aware of this anomaly. The anomaly is usually associated with unilateral renal agenesis and other renal anomalies [3-5].

## Case report

A 31-year-old man was worked up for a symptomatic, reducible right inguinal hernia. There were no urinary or abdominal symptoms. His history was significant only for an uneventful, left open inguinal hernia repair one year previously at our center. No duplication of vas deferens or any other anomaly was observed. On examination, the patient had an easily reducible, moderate-sized, right inguinal hernia with normal testes. Ultrasound examination of the abdomen and pelvis did not reveal any abnormality.

**Figure 1:**
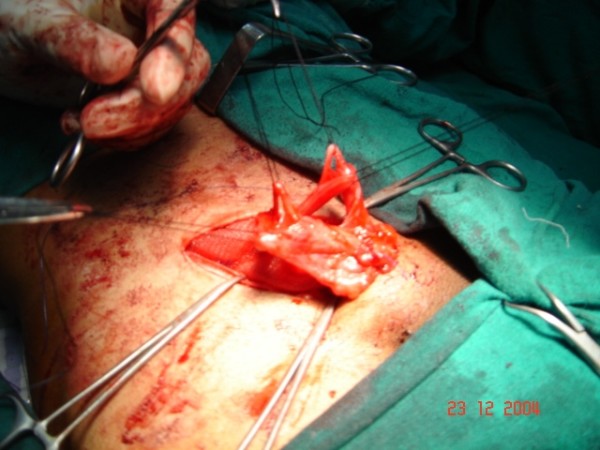
**The hernial sac has been dissected free from the two vas deferens (taken on slings)**.

**Figure 2:**
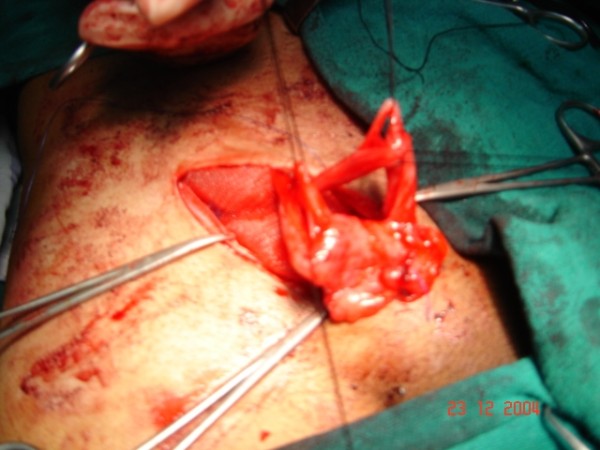
**The cord contents are seen with duplication of vas deferens**. The prolene mesh has been sutured in to place.

At operative exploration, an indirect hernia was identified. During dissection of the hernia sac from adjacent cord structures, two separate, equal-sized vas deferens at a distance of 2 cm from each other, posteromedial to the hernial sac were isolated (fig. 1, fig. 2). Intra-operative examination of the scrotum demonstrated a single normally located testicle and epididymis. Both vas deferens were communicating with the epididymis. Care was taken to keep both structures intact during high dissection of the hernia sac and while suturing the mesh into place. The patient tolerated the procedure well and had an uncomplicated postoperative course. Postoperative ultrasound examination of the abdomen did not reveal any other anomaly. Follow up of two years is uneventful

## Discussion

Duplication of vas deferens is a rare congenital anomaly for which the overall incidence in the general population is estimated to be less than 0.05% [2-4]. About 1% of boys have an absent vas, which may be accompanied by renal agenesis[4]. Abnormalities of the vas deferens and ureter are explained embryologically using the concept of the proximal vas precursor (PVP). The PVP is the segment along mesonephric duct that occupies an intermediate position between the upper and common mesonephric ducts(CMD). It differentiates in to the vas deferens and seminal vesicles. The more caudal CMD forms a ureteral bud that will grow to contact the metanephric blastema resulting in the kidney and collecting system[4]. Reported cases of so-called double vas or true duplication of vas involve renal dysgenesis and an ectopic ureter simulating a double vas [2-4]. Duplication of the PVP presumably gives rise to partial duplication of the vas deferens at the level of the inguinal canal [3].

Unilateral duplication of vas deferens is rare and may predispose to Injury to the vas deferens during hernia surgery [1-8]. A duplicated vas deferens may be associated with other congenital abnormalities such as ipsilateral renal agenesis and cystic fibrosis[6,7]. Anomalies of vas deferens can be categorized as absence, duplication, ectopia, hypoplasia and diverticulum.

Injury to the vas is a recognized complication during hernia surgery that may lead to infertility, chronic pain, and spermatic granulomas[8,12]. Identification of the vas deferens during exploration of the cord for a hernia sac therefore is considered mandatory in order to prevent its iatrogenic injury. In patients with a unilateral duplicated vas deferens, the anatomic variant may not be recognized, resulting in increased chances of intra-operative injury and subsequent complications. Although the duplicated vas may be smaller or incomplete, the complications associated with its injury are the same i.e. scarring of the vas deferens with obstruction, spermatic granulomas, and chronic pain. With a large number of inguinal hernia repairs performed annually, surgeon's awareness of this possible anatomic anomaly is necessary in order to minimize the risk of complications [8-14].

## Conclusion

Duplication of vas deferens is rare with only few cases reported in the world literature. A hernia surgeon needs to be aware of this anomaly in order to avoid injury to this vital structure. Routine identification of vas deferens and awareness about this anomaly during surgery is therefore strongly recommended.

## Consent

A written informed consent was taken from the patient regarding publication of this case report in any journal.

## Competing interests

The authors declare that they have no competing interests.

## Authors' contributions

CM was the chief operating surgeon, RK, YK, MT were the surgical assistants.

## References

[B1] Gray's Anatomy Pick, Howden Commemorative edition 1901 New York: Bounty Books 102117794767

[B2] Erdemir F Parlaktas BS Adem Yasar Uluocak N Duplicated Vas Deferens: A rare congenital abnormality Kaohsiung J Med Sci 2008 24 4 210 211 10.1016/S1607-551X(08)70119-0 18424358 PMC11918087

[B3] Atug F Turkeri L Partial duplication of the vas deferens at the level of inguinal canal Int J Urol 12 8 773 775 16174056 10.1111/j.1442-2042.2005.01147.x

[B4] Binderow SR Shah KD Dolgin SE True duplication of the vas deferens Journal Of Pediatric Surgery 1993 28 2 269 70 10.1016/S0022-3468(05)80293-3 8437095

[B5] Amelar RD Dubin L Walsh PC Embryology, anatomy and physiology of the male reproductive tract Male Infertility 1977 Philadelphia: W. B. Saunders 26

[B6] Vohra S Morgentaler A Congenital anomalies of the vas deferens, epididymis and seminal vesicles Urology 1997 49 313 10.1016/S0090-4295(96)00433-5 9123691

[B7] Damle S Cothren C C Moore E E Kim FJ Double Trouble: Duplication of Vas Deferens Encountered During Inguinal Hernia Repair J Am Coll Surg 2005 201 1 141 10.1016/j.jamcollsurg.2004.12.031 15978455

[B8] Mathe CP Dunn G Double vas deferens associated with solitary kidney J Urol 1948 59; 461 18903559 10.1016/S0022-5347(17)69398-4

[B9] Silich RC McSherry GK Spermatic granuloma: An uncommon complication of the tension-free hernia repair Surg Endosc 1996 10 537 539 10.1007/BF00188403 8658335

[B10] Mysorekar VR Accessory vas deferens a case report Br J Urol 1976 48 1; 82 10.1111/j.1464-410X.1976.tb02749.x 1268471

[B11] Yeats WK Blandy JP Urology 11 Oxford: Blackwell 1279

[B12] Carr R Private medical clinic. Blyth Apparent Bilateral Duplication of the Vas Deferens British Journal of Urology 1993 71 354 360 10.1111/j.1464-410X.1993.tb15958.x 8477322

[B13] Sheynkin YR Hendin BN Schlegel PN Goldstein M Micro-surgical repair of iatrogenic injury to the vas deferens J Urol 1998 159 139 141 10.1016/S0022-5347(01)64036-9 9400456

[B14] LeBlanc KA Complications associated with the plug-and-patch method of inguinal herniorrhapy Hernia 2001 5 135 138 10.1007/BF01576164 11759798

